# Characterization and Validation of a Human 3D Cardiac Microtissue for the Assessment of Changes in Cardiac Pathology

**DOI:** 10.1038/s41598-018-28393-y

**Published:** 2018-07-05

**Authors:** Caroline R. Archer, Rebecca Sargeant, Jayati Basak, James Pilling, Jennifer R. Barnes, Amy Pointon

**Affiliations:** 1 Safety and ADME Translational Sciences, Drug Safety and Metabolism, IMED Biotech Unit, AstraZeneca, Cambridge, CB4 0WG UK; 2 Pathology Sciences, Drug Safety and Metabolism, IMED Biotech Unit, AstraZeneca, Cambridge, CB4 0WG UK; 3 Discovery Sciences, IMED Biotech Unit, AstraZeneca, Cambridge, CB4 0WG UK

## Abstract

Pharmaceutical agents despite their efficacy to treat disease can cause additional unwanted cardiovascular side effects. Cardiotoxicity is characterized by changes in either the function and/or structure of the myocardium. Over recent years, functional cardiotoxicity has received much attention, however morphological damage to the myocardium and/or loss of viability still requires improved detection and mechanistic insights. A human 3D cardiac microtissue containing human induced pluripotent stem cell-derived cardiomyocytes (hiPS-CMs), cardiac endothelial cells and cardiac fibroblasts was used to assess their suitability to detect drug induced changes in cardiac structure. Histology and clinical pathology confirmed these cardiac microtissues were morphologically intact, lacked a necrotic/apoptotic core and contained all relevant cell constituents. High-throughput methods to assess mitochondrial membrane potential, endoplasmic reticulum integrity and cellular viability were developed and 15 FDA approved structural cardiotoxins and 14 FDA approved non-structural cardiotoxins were evaluated. We report that cardiac microtissues provide a high-throughput experimental model that is both able to detect changes in cardiac structure at clinically relevant concentrations and provide insights into the phenotypic mechanisms of this liability.

## Introduction

Cardiovascular diseases are currently recognized as the leading cause of death in the world, in 2013 accounting for >17 million deaths^1^. Subsequently, large numbers of the population have preexisting cardiovascular disease. This, combined with a large number of pharmaceuticals causing unintended cardiovascular toxicities, puts an additional burden on already compromised hearts^2–4^.

Small-molecule compounds continue to be developed and prescribed, improving life expectancy across several disease indications. Over recent years significant progress in antineoplastic compounds, include tyrosine kinase inhibitors and proteasome inhibitors, has increased cancer patient survival. These agents significantly reduce cancer cell survival, proliferation and migration but are all associated to some degree with cardiotoxicities. This ultimately resulting in the development of chronic heart failure, myocardial infarction, myocarditis and/or cardiomyopathy^5–7^. As cancer is progressively becoming a treatable condition, it is increasingly important to reduce these deleterious side effects. This is particularly pertinent as currently cardiotoxicity is recognised as a leading cause of long-term morbidity and mortality among cancer survivors^8^. This, combined with the prevalence of cardiovascular disease, requires improved approaches to allow new compounds to be discovered and developed without these liabilities, impacting the clinical utility and patient outcome.

Drug-induced cardiotoxicity can affect all components and functions of the cardiovascular system, either directly or indirectly, and can be functional and or structural (morphological) in nature, as previously defined by Laverty *et al*.^9^. The interactive sequence in which both structural and functional cardiotoxicity manifests is compound specific^10^. Structural cardiotoxicity can be viewed as a continuum progressing from degeneration, necrosis leading to inflammatory changes and fibrosis, affecting multiple cardiac cell types. Throughout this process the ability of the heart to contract and function is highly dependent on the number, severity and distribution of cells involved^11^. For example, extensive fibrosis can affect myocardial compliance and contractility resulting in a cardiac functional decline. Conversely, ultrastructural abnormalities may not initially modulate cardiac function until the burden of cardiomyocyte loss and/or remodelling increases. These later changes may not result in a functional decline for many years due to the normalisation of function via compensatory mechanisms. In addition to structural modifications resulting in functional consequences, prolonged changes in haemodynamics, particularly increases in heart rate are associated with the subsequent development of changes in cardiac structure^12^.

Preclinically, various simple *in vitro* approaches and animal models are used to identify cardiac liabilities before a compound enters clinical development. Currently, *in vitro* approaches typically range from single organ assays e.g.: the isolated whole heart from preclinical species, isolated ventricular cardiomyocytes from preclinical species to cell lines over expressing single ion channels^13–15^. These approaches have limitations when detecting cardiotoxicity, primarily due to the length of time these models can remain viable, bearing in mind the development of chronic heart failure and cardiomyopathy can take days to months/years to develop clinically^16,17^. The elements of cardiotoxicity they are able to predict are predominately focused on functional changes and cardiomyocytes, neglecting the influence of non-myocytes and morphological damage (structural cardiotoxicity). Combined with a lack of molecular understanding responsible for changes in cardiac morphology, these liabilities are often only first detected in repeat dose animal studies, late in discovery, once chemical choice is limited^11^. The development of human stem cell derived cardiomyocytes has offered a new opportunity to detect changes in cardiac structure of novel agents earlier in the discovery process, allowing these liabilities to be not only identified but designed out of the chemistry, allowing new compounds without these effects to enter clinical development^18^. However, approaches currently applied to detect structural cardiotoxicity use human embryonic stem cell derived cardiomyocytes (hESC-CMs), which have associated ethical concerns, use ‘cardiac like’ cell lines e.g. rat H9C2 cells^19^ which lack key characteristics representative of cardiomyocytes or use cells from preclinical species^20^. Additionally, none of these models take into consideration the role of non-cardiomyocytes. This is a limitation when the proportion of non-cardiomyocytes in the myocardium, which contributes to approximately 70% of the cell mass, is taken into consideration^21^. These non-cardiomyocytes have an important role in cardiac physiology and functionality^21–24^. The majority of non-cardiomyocytes in the heart are cardiac fibroblasts and endothelial cells. Cardiac fibroblasts are known to contribute to mechanotransduction, electrical conduction and synthesis of extracellular matrix^21,22,25,26^. Whereas endothelial cells influence cardiac metabolism, contractile performance, survival and rhythmicity^27^. These physiological changes occur due to bidirectional cross talk between non-cardiomyocytes and cardiomyocytes via auto and paracrine signalling^28–30^. Taking together the physiological role of these two major non-cardiomyocyte cell types, it is logical to suggest these cells contribute to the development and/or response to structural cardiotoxicity. This is particularly pertinent when considering the dynamic relationship between cardiac contractility, which both fibroblasts and endothelial cells influence, and the progression to morphological damage. Indeed, in recent years these cell types have been implicated in doxorubicin and sunitinib-induced structural cardiotoxicity^31–33^.

Here, we applied and characterised a 3D cardiac microtissue containing human induced pluripotent stem cell-derived cardiomyocytes (hiPS-CMs), cardiac fibroblasts and cardiac endothelial cells, combined with 15 FDA approved antineoplastic, antiarrhythmic, antifungal and antipsychotic compounds all associated with structural cardiotoxicity and 14 clinical compounds with no association with structural cardiotoxicity. We used morphological and biomarker assessment, high content biology (mitochondrial membrane potential (ΔΨm) and endoplasmic reticulum (ER) integrity) and cellular viability assays (ATP depletion) to establish an approach to detect structural cardiotoxicity and provide phenotypic mechanistic fingerprints into these liabilities.

## Results

### Cellular and morphological assessment of cardiac microtissues

Cardiac microtissues were formed using 384 well round-bottom ultra-low adhesion plates combining hiPS-CMs, human cardiac microvascular endothelial cells and human cardiac fibroblasts. Following 14 days in culture, spontaneously beating cardiac microtissues were formed. All experiments were conducted on microtissues between 14 and 21 days in culture in a blinded manner.

**Figure 1:**
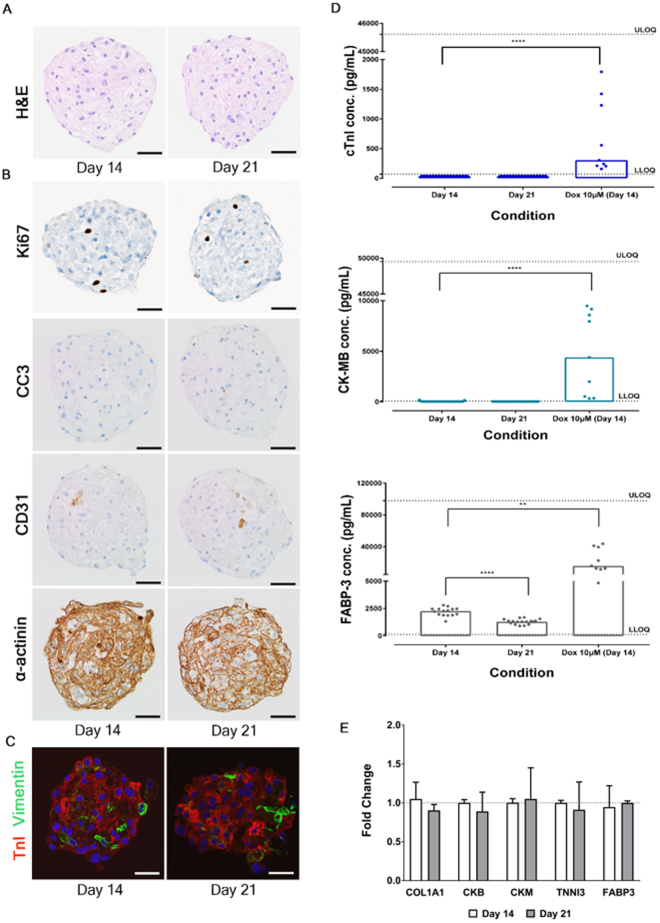
Cellular composition, morphology and cardiac biomarker expression are stable in microtissues over time at Day 14 and Day 21. (**A**) Representative images of haematoxylin and eosin (H&E) stained microtissues. (**B**) Representative immunohistochemistry (IHC) images of microtissues immunostained with Ki67, cleaved caspase-3 (CC3), CD31, and alpha-actinin (α-actinin). (**C**) Representative immunofluorescence (IF) images of microtissues dual labelled with troponin I (TnI) and vimentin. All morphology data evaluated a minimum of 15 microtissues per time point. Scale bar represents 50 µm. (**D**) Soluble biomarker data showing unstimulated basal levels and stimulated (10 µM doxorubicin) of cardiomyocyte structural proteins cardiac troponin I (cTnI), Creatine Phosphokinase-MB (CK-MB) and Fatty Acid Binding Protein-3 (FABP-3) released in media on Day 14 and Day 21. Data represents individual and median ± SEM (unstimulated: *n* = >34, on 3 independent cardiac microtissue preparations, stimulated: *n* = 3, on 3 independent cardiac microtissue preparations). (**E**) qRT-PCR data showing expression of cardiomyocyte biomarker genes and collagen 1 A up to Day 21 (grey bar), relative to the Day 14 (open bar) control in cardiac microtissues. Each bar represents the mean ± SEM (*n* = 308 on 3 independent cardiac microtissue preparations). **p < 0.01, ****p < 0.0001, LLOQ; lower level of quantification, ULOQ; upper level of quantification, dox; doxorubicin.

A comprehensive assessment of the cellular composition and morphological integrity, via haematoxylin and eosin (H&E) staining, immunohistochemistry of key viability and cell type specific markers, assessment of soluble cardiac biomarkers and gene expression analysis was conducted over microtissues in culture for 14 to 21 days. H&E staining confirmed slight cytoplasmic vacuolation and/or eosinophilic protein globules, these were predominately in cardiomyocytes, at a low incidence. Small areas of acellular eosinophilic proteinaceous material was also observed. Despite these findings no significant morphological abnormalities were detected, no differences were observed over time (i.e. between day 14 and day 21) and a lack of a necrotic core in all cardiac microtissues examined was confirmed (Fig. 1A). Further assessment of cell viability, in particular apoptosis via immunohistochemistry of cleaved caspase-3 (CC3), revealed slight variability within the microtissues, but all positive staining was of low levels with only individual cells or small cell clusters showing morphological features consistent with apoptosis or CC3 immunopositivity (Fig. 1B). Additionally, Ki-67 staining revealed a low level of staining which was consistent throughout the experimental time frame (Fig. 1B). Having established that the microtissues were morphologically intact, we confirmed the presence and integrity of each cell type (cardiomyocytes, endothelial cells and fibroblasts) via immunohistochemical detection of key structural proteins. Alpha actinin and cardiac troponin I (cTnI) were used as specific cardiomyocyte markers, CD31 as a marker of endothelial cells and vimentin as a marker of fibroblasts. All expected cell types were positively identified within the microtissue and importantly the approximate proportions of each cell type was consistent with the anticipated starting cell ratios of 4:2:1 (cardiomyocytes:fibroblasts:endothelial cells). This, along with a consistently low level of Ki-67 staining, a marker of an active cell cycle state, demonstrates an inherently low level of both cell proliferation and loss of cell viability in untreated microtissues (Fig. 1B and C). Moreover, no differences were observed in cellular composition between microtissues cultured for 14 and 21 days. Cardiomyocytes abundantly expressed the sarcoplasmic proteins, alpha actinin and cTnI. The pattern of expression delineated well defined anastomosing, filamentous structures within the cardiomyocytes (presumptive cardiac sarcomeres; Supplementary Fig. 1). Fibroblasts and endothelial cells stained positively for vimentin and CD31, respectively, at a proportion approximately relative to the starting cell numbers of each cell type. Additionally, dual immunofluorescent staining for cTnI and vimentin identified two largely distinct populations of cells with limited cells co-expressing both proteins (Fig. 1C). Due to the lack of specificity of vimentin as a marker of fibroblasts, to further confirm the stability of the fibroblasts in microtissues cultured for 14 and 21 days, collagen 1 A gene and protein expression was determined, no significant increase or gross morphological differences were observed over these time points (Fig. 1E and Supplementary Fig. 1). To further complement this morphological assessment, known soluble cardiac biomarkers (cTnI, creatine phosphokinase muscle/brain (CK-MB) and fatty acid binding protien-3 (FABP-3)) were monitored over the same time course (Fig. 1D). These biomarkers are released from cardiomyocytes upon damage, and significantly elevated levels would be suggestive of morphological damage. To enable basal levels of these proteins to be distinguished from those associated with damage, a known structural cardiotoxin, doxorubicin at a clinically relevant concentration was included as a positive control. Both cTnI and CK-MB were detected below the lower level of quantification on day 14 and 21 of culture. 10 µM doxorubicin significantly increased release 40.5 and 268.5 fold for cTnI and CK-MB, respectively. FABP-3 levels were detectable on day 14 and 21 of culture, however, 10 µM doxorubicin increased release by 18.2 fold (Fig. 1D). This data further supports the lack of morphological damage observed by H&E, immunohistochemistry and immunofluorescence. To ensure that the absence of cTnI and CK-MB release was due to a lack of cardiomyocyte damage and not due to the lack of expression, gene expression of these biomarkers was assessed (Fig. 1E). The presence of each biomarker was confirmed, with gene expression being comparable between days 14 and 21 in culture, ratifying that the lack of release of cTnI (TNNI3) and CK-MB (CKB and CKM) reflects a lack of cardiomyocyte damage. This combined cellular composition, morphology, biomarker and gene expression data confirms these microtissues are a suitable model system to evaluate cardiac biology including drug-induced changes in cardiac structure. In addition, there was no apparent effect of increased culture duration (day 14–21) on either cellular degeneration or necrosis.

### Drug-induced morphological and viability changes in cardiac microtissues

**Figure 2:**
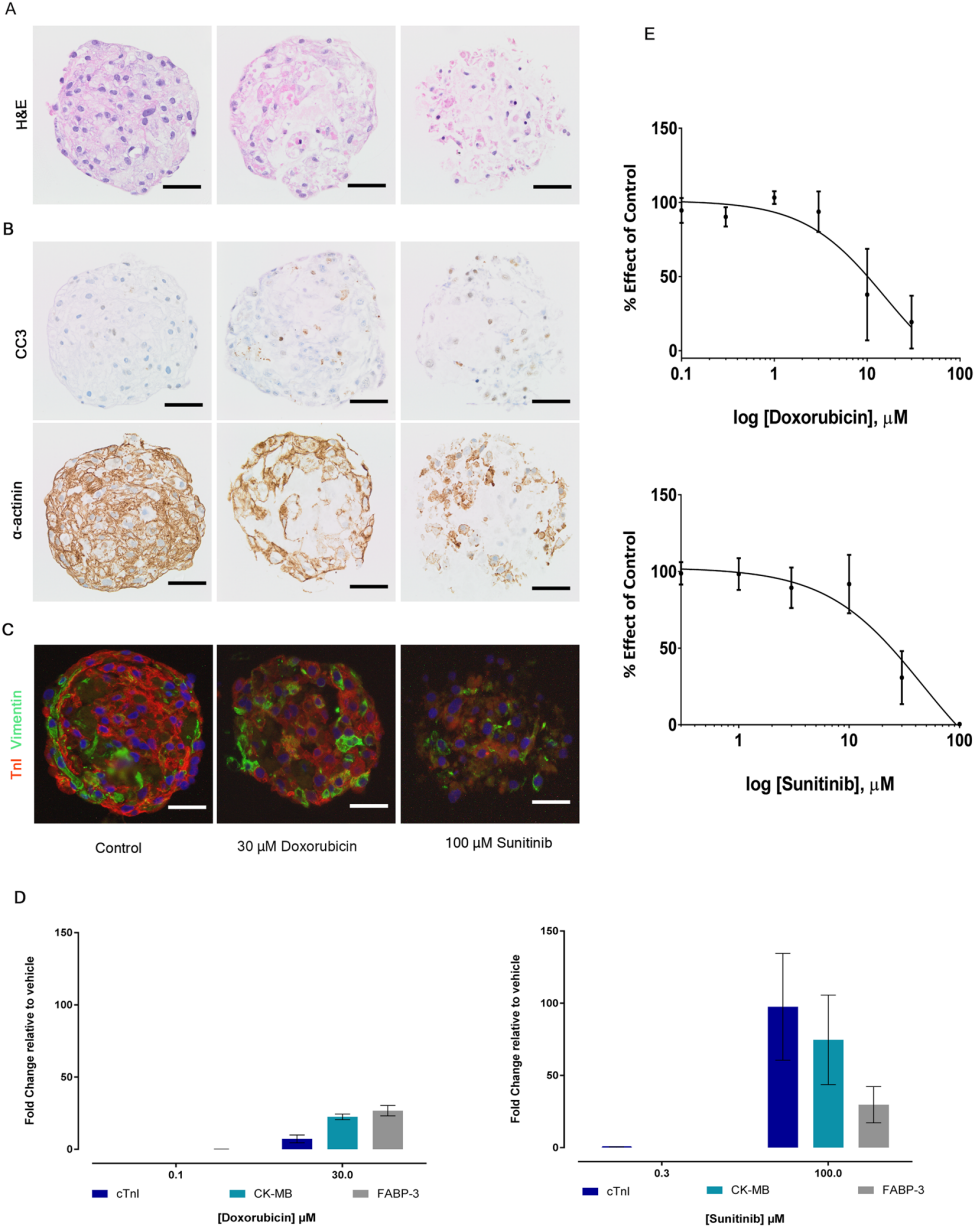
Treatment with known structural cardiotoxins for 24 hours results in significant histopathological changes and loss of key structural proteins. (**A**) Representative images of H&E stained microtissues showing degenerative histopathological changes. (**B**) Representative IHC images showing an increase in CC3 expression and reduced expression of the cardiomyocyte structural protein α-actinin in drug treated microtissues. (**C**) Representative IF images of microtissues dual labelled with troponin I (TnI) and vimentin showing the reduced expression. All morphology data evaluated a minimum of 15 microtissues per treatment. (**D**) Soluble biomarker data (blue: cTnI, green: CK-MB and grey: FABP-3) following 0.3 and 100 µM sunitinib and 0.1 and 30 µM doxorubicin (*n* = 3 on 3 independent cardiac microtissue preparations, mean ± SD). (**E**) Concentration-effect curves showing the effect of sunitinib and doxorubicin on cell viability (ATP depletion) in cardiac microtissues after 24 hours (*n* = 8 on 4 independent cardiac microtissue preparations, mean ± SEM). Scale bar represents 50 µm.

To determine whether cardiac microtissues were a suitable model to evaluate drug-induced effects on cardiac structure, we assessed the morphological integrity, cellular composition, release of cardiac biomarkers and viability of these microtissues following two known antineoplastic structural cardiotoxins, the anthracycline, doxorubicin and the tyrosine kinase inhibitor, sunitinib. Under these conditions, microtissues displayed significant histopathological changes and loss of key structural proteins in a concentration dependant manner. These morphological changes were accompanied by increases in released cardiac biomarkers (cTnI, CK-MB, FABP-3) in culture media (Fig. 2 and Supplementary Fig. 2). Treatment with 100 µM sunitinib for 24 hours resulted in a clear reduction in overall cellularity. The remaining cells in the microtissue displayed significant degenerative changes, with the occurrence of occasional condensed pyknotic nuclei with hypereosinophilic cytoplasmic accumulations, and an increase in the amount of acellular eosinophilic debris interspersed between the residual cells. These changes were accompanied with a reduction in alpha actinin, cTnI and vimentin protein expression and increased CC3 indicating a loss of structural integrity and elevated levels of apoptosis, respectively. Concurrent to these changes, cTnI, CK-MB and FABP-3 were elevated in culture media at 19, 92.3 and 10.3-fold, respectively, following 100 µM sunitinib. Compared to sunitinib, doxorubicin exposure induced similar morphological and cardiac biomarker changes, but of a lower severity (Fig. 2D). Accompanying these gross morphological changes, cellular viability (ATP depletion) levels were reduced in a concentration dependent manner with an IC_50_ value of 46.24 µM (95% confidence interval (CI), 9.13 to 234.2) and 15.5 µM (95% CI 1.12 to 214) for sunitinib and doxorubicin, respectively (Fig. 2E). These results suggest that cardiac microtissues respond in terms of morphology and viability following clinical structural cardiotoxins, signifying they are a suitable model system to detect changes in cardiac structure at the morphological level.

### Impact of structural cardiotoxins on hiPS-CMs, cardiac endothelial cells and cardiac fibroblasts

As these cardiac microtissues appear to be a suitable model to investigate structural cardiotoxicity, we wanted to understand the relative contribution and importance of each of the three cell constituents. To provide some insights, hiPS-CMs, cardiac endothelial cells and cardiac fibroblasts were exposed to three antineoplastic structural cardiotoxins, doxorubicin, sunitinib and lapatinib for 24 and 72 hours. Under these conditions, each cell type displayed changes in cellular viability (ATP depletion) and subcellular organelles (mitochondria and ER). However, the relative degree of change differed between the three cell types, suggesting that cardiomyocytes and non-cardiomyocytes are modulated to varying degrees (Supplementary Fig. 3 and Supplementary Table 1). For example, lapatinib preferentially reduced cellular viability (ATP depletion) in cardiac endothelial cells compared to cardiac fibroblasts and hiPS-CMs with an IC_50_ values of 3.07 µM (95% CI 2.77), 11.04 µM (95% CI 3.51) and >100 µM, respectively. These results suggest that lapatinib induces endothelial cell damage which subsequently promotes cardiomyocyte and cardiac fibroblast dysfunction. Similar results were obtained following sunitinib treatment. Whereas, doxorubicin preferentially modulated both cardiac endothelial cells and cardiac fibroblasts at 24 hours. Taken together this data supports the inclusion of all three cell types in the cardiac microtissue.

### High-throughput imaging and cell viability analysis of antineoplastic structural cardiotoxins in cardiac microtissues

**Figure 3:**
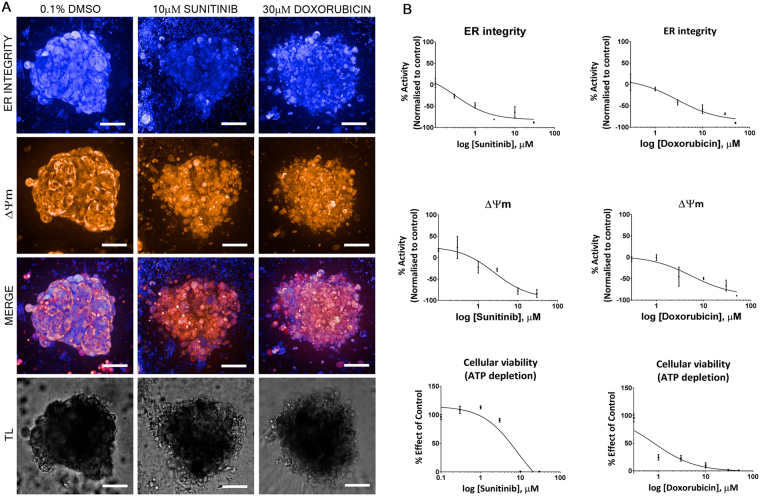
Development of high-throughput imaging and cellular viability assays for the detection of structural cardiotoxicity. (**A**) Representative transmitted light and fluorescent image of cardiac microtissues treated with vehicle (0.1% DMSO (v/v)), 10 µM sunitinib or 30 µM doxorubicin for 72 hours. Cardiac microtissues stained with ER tracker (ER integrity) or TMRM (ΔΨm). Scale bar represents 100 µm (*n* = 4 on 3 independent cardiac microtissue preparations). (**B**) Typical non-cumulative concentration-effect curves generated for ER Integrity, ΔΨm and cellular viability (ATP depletion) of cardiac microtissues treated with either sunitinib or doxorubicin (*n* = 4 on 3 independent cardiac microtissue preparations, mean ± SEM). TL; transmitted light, ΔΨm; mitochondrial membrane potential.

As standard histopathology of these cardiac microtissues was shown to detect changes in cardiac structure in a low throughput manner and the relative importance of non-cardiomyocytes has been determined, a novel higher throughput approach was required to allow application of these cardiac microtissues to support the detection and risk assessment of drug-induced changes in cardiac structure during drug discovery. To identify the most appropriate assays to investigate structural cardiotoxicity *in vitro*, a panel of live cell fluorescent imaging assays were developed and utilised, namely ΔΨm, ER integrity and transmitted light (Fig. 3A). These parameters allow the assessment of two major organelles along with a marker of cellular viability. Despite the limited molecular understanding of structural cardiotoxicity, it is logical to presume that both the mitochondria and ER are involved either as a primary or secondary effect. These imaging parameters were combined with an assessment of cellular viability (ATP depletion); following treatment with the anthracycline, doxorubicin and the tyrosine kinase inhibitor, sunitinib. As structural cardiotoxicity can develop over the time course of days to months clinically, we exposed the cardiac microtissues to each compound for 72 hours. Measuring several parameters simultaneously is mechanistically insightful by identifying the parameter modulated at the lowest concentration formulating a unique fingerprint for each compound. 10 µM sunitinib preferentially reduced ER integrity, preceding visual decreases in ΔΨm and transmitted light, respectively. Whereas, following 30 µM doxorubicin all imaging parameters where modulated simultaneously. These multichannel images were further quantified by automated image analysis algorithms, allowing concentration-effect curves to be generated and IC_50_ values to be calculated (Fig. 3B). This allows the identification of quantitative mechanistic fingerprints, such an approach confirmed the fingerprint for sunitinib, but for doxorubicin the combination of a cell viability assessment (ATP depletion) in combination with these imaging parameters identified a decrease in cell viability that was preceded by a decrease in both ER integrity and ΔΨm. These results suggest that a combination of imaging parameters and assessment of cellular viability in cardiac microtissues can be applied in a high-throughput manner to detect changes in cardiac structure.

**Figure 4:**
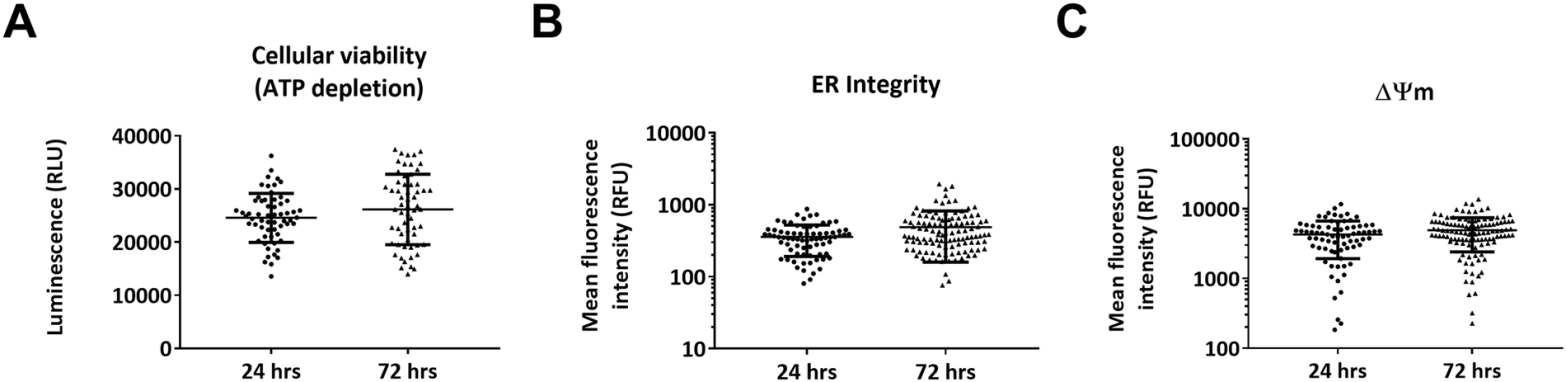
Imaging and cellular viability parameters are stable over 72 hours. Comparison of (**A**) Cellular viability (ATP depletion), (**B**) ER integrity or (**C**) ΔΨm average fluorescence intensity at Day 14 + 24 hours versus Day 14 + 72 hours in cardiac microtissues (*n* ≥ 20 on >3 independent cardiac microtissue preparations, mean ± SD). Statistics by two-tailed *t-*test, no statistical significance observed for cellular viability and ΔΨm (*P* > 0.05). A significant difference between time points was observed for ER integrity parameter (*P* ≤ 0.01). ΔΨm; mitochondrial membrane potential.

Prior to wider pharmacological validation of this approach, the variability of each quantifiable parameter was assessed over the experimental timeframe. Microtissues were assessed following 14 days in culture and subsequently 72 hours later (Fig. 4). This ensures any negative changes in parameters at 72 hours can be directly attributed to pharmacological activity. Cellular viability was found to be 24570 ± 2681 (RLU) and 26150 ± 6031 (RLU) (mean ± SD, *n* = 3), at day 14 and 72 hours later, respectively. ΔΨm was found to be 4286 ± 2365 (average fluorescence intensity) 4917 ± 2508 (average fluorescence intensity) (mean ± SD, *n* = 4), at day 14 and 72 hours later, respectively. ER integrity was found to be 357 ± 166 (average fluorescence intensity) and 489 ± 330 (average fluorescence intensity) (mean ± SD, *n* = 4), at day 14 and 72 hours later, respectively. These data for cellular viability and ΔΨm indicated no statistically significant differences over the period of the experiment (72 hours). However, a significant increase was observed for ER integrity (*P* < 0.01).

### Cardiac microtissues are a sensitive model for the assessment of structural cardiotoxicity

The potential of this approach to detect a pharmacologically diverse panel of 15 FDA approved antineoplastic, antiarrhythmic, antifungal and antipsychotic compounds all associated with structural cardiotoxicity were assessed in quadruplicate on at least three independent occasions. This approach was capable of determining an IC_50_ value in at least one parameter for all structural cardiotoxins evaluated (Supplementary Table 2). In addition, we found all three topoisomerase II inhibitors, idarubicin, mitoxantrone and doxorubicin, were in the top five most potent compounds evaluated, with mean lowest IC_50_ values in at least one parameter of 0.03 (cellular viability), 0.29 (cellular viability) and 0.18 µM (ΔΨm), respectively. Sunitinib was the most potent tyrosine kinase inhibitor with a mean IC_50_ value of 0.39 µM (ER integrity). Whereas the least potent compounds were cyclophosphamide and isoproterenol with a mean IC_50_ value of 54.44 (cellular viability) and 59.25 µM (ΔΨm), respectively. Dasatinib was the least potent tyrosine kinase inhibitor with a mean IC_50_ value of 27.14 µM (cellular viability).

**Figure 5:**
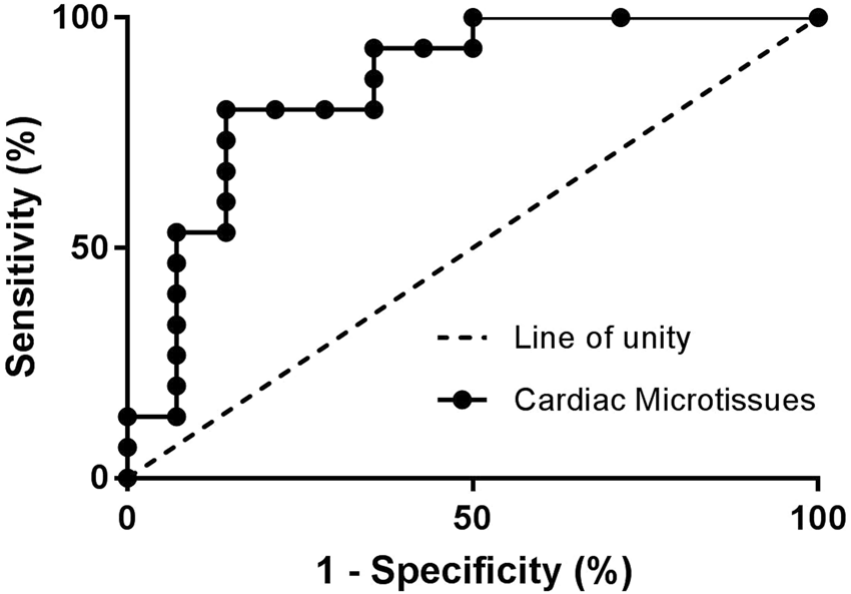
Cardiac microtissues are predictive of structural cardiotoxicity. The lowest geometric mean obtained (IC_50_) for cellular viability (ATP depletion), ER integrity or ΔΨm were subjected to ROC curve analysis. The dashed line of unity shows the results of random assignments of structural cardiotoxicity (*n* > 4 on 3 independent cardiac microtissue preparations).

To ensure that this approach would offer both sensitivity and specificity for the detection of changes in cardiac structure, 14 clinical compounds with no association with structural cardiotoxicity were also included with the positive compounds (Supplementary Table 2). The most sensitive parameter potency value (ATP depletion, ΔΨm, ER integrity) for each compound was used to produce a receiver operator curve (ROC) curve (Fig. 5). ROC curves are an exhaustive approach to examine paired true-positive (sensitivity) and false-positive rates (1-specificity) for all possible thresholds^34^. This allows the outcome of compound effects to be compared to the known effects *in vivo*, in this case structural cardiotoxicity. Use of ROC curves allows appropriate cut-off levels to be selected to judge if a compound is active or inactive in the assay. We found that an IC_50_ value of 10 μM or below gave optimal sensitivity and specificity. Using this threshold, the sensitivity (ability to detect true-positive compounds) of this approach is 73%, whereas the specificity (ability to detect true-negative compounds) of this approach is 86%.

**Figure 6:**
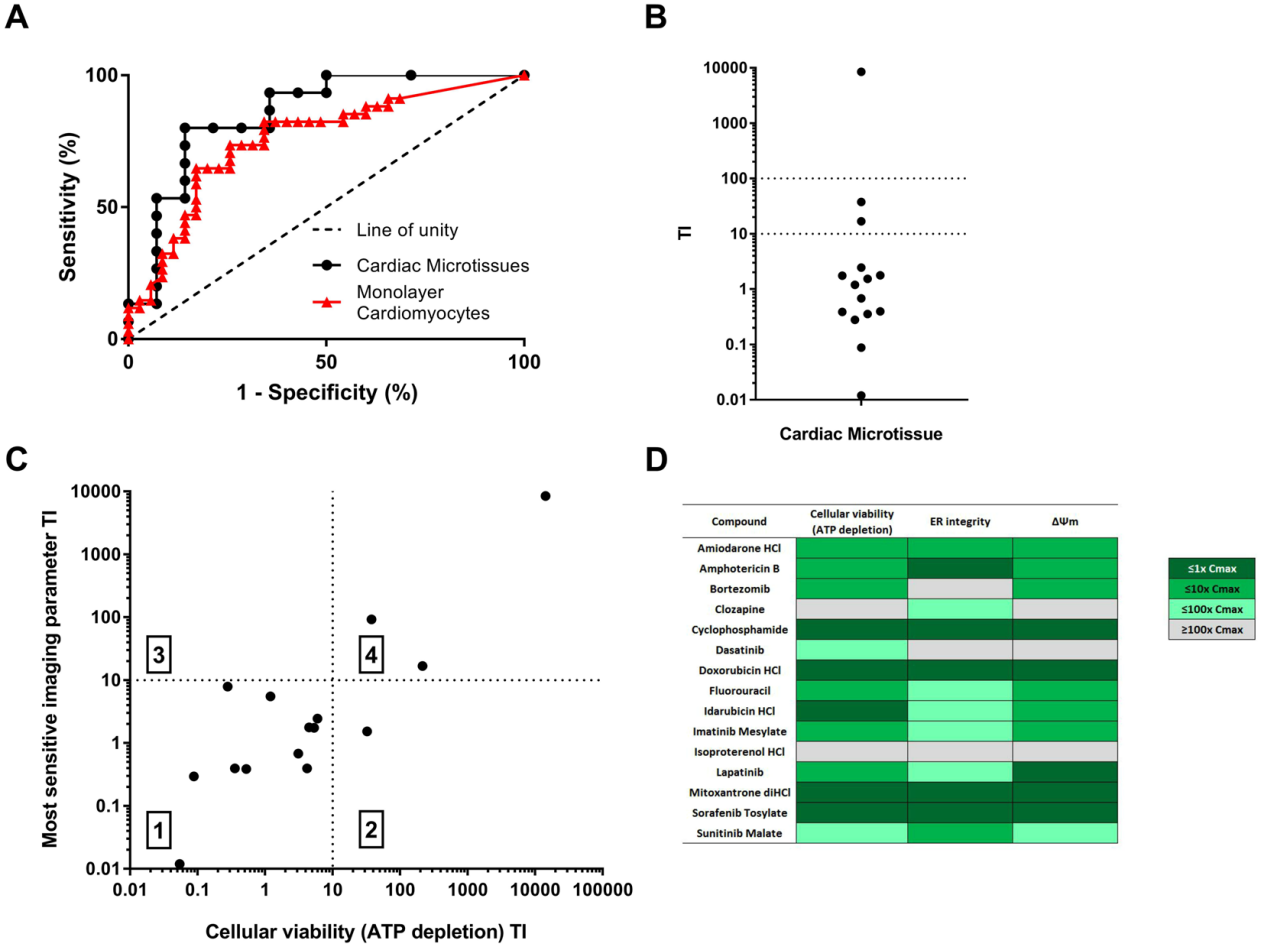
Imaging and cellular viability parameters allow structural cardiotoxicity to be detected at therapeutically relevant concentrations and facilitates molecular phenotypic insights. (**A**) The lowest geometric mean obtained (IC_50_) for cellular viability (ATP depletion), ER integrity or ΔΨm was subjected to ROC analysis. Black circles: cardiac microtissues, red triangles: monolayer cardiomyocytes. The dashed line shows the results of random assignments of structural cardiotoxicity (*n* > 4 on 3 independent cardiac microtissue preparations). (**B**) Potency values (*n* > 4 on 3 independent cardiac microtissue preparations) obtained in cardiac microtissues were normalized to the total Cmax of each compound and expressed as a ratio (TI). A value of one or below indicates the IC_50_ for an assay was either at or below Cmax concentrations, whereas a value >1 indicates the IC_50_ for an assay was above Cmax concentrations. The plot shows the TI which compounds were detected at. The dotted lines represent hundred times and ten times Cmax. (**C**) Distribution maps between cellular viability (ATP depletion) and imaging parameters in cardiac microtissues. The geometric mean IC_50_ (*n* > 4 on 3 independent cardiac microtissue preparations) obtained in either ATP depletion or an imaging parameter (ER integrity or ΔΨm) were normalized to Cmax (TI). The plot shows the number of compounds detected by both, ATP depletion and an imaging parameter, quadrant 1, an imaging parameter alone, quadrant 2, ATP depletion alone, quadrant 3, or compounds not detected at or below ten times Cmax concentrations, quadrant 4. (**D**) Classification of compounds into thematic mechanisms. Compounds were classified into categories based on the pattern of response in each assay in relation to the Cmax concentration. This allowed compound effects to be separated into those occurring at 1, 10, and 100 times Cmax concentrations.

To determine if the cardiac microtissue, with 3D morphology and three major cardiac cell types, differed in terms of pharmacological responses, to monolayer approaches, data was compared to that obtained in monolayer cardiomyocytes, namely hESC-CMs. Overall, data compared favourably, with the ROC curve for the cardiac microtissue being slightly shifted to the left of the hESC-CM curve (Fig. 6A). The resulting sensitivity and specificity values obtained in hESC-CMs are 74% and 74%, respectively^18^. This indicates comparable sensitivity and improved specificity of the cardiac microtissue compared to monolayer approaches, illustrating that cardiac microtissues are an applicable model system.

### Translation of findings at therapeutically relevant concentrations

The outcome of a drug being either toxic or non-toxic is determined by exposure. Consequently, it is important to consider therapeutically relevant maximum total plasma concentrations (Cmax) when investigating drug-induced toxicity. We questioned whether this approach can detect changes in drug-induced cardiac structure at relevant therapeutic concentrations. The lowest geometric mean IC_50_ (>*n* = 3) obtained in either ΔΨm, ER integrity (imaging parameters) or cellular viability (ATP depletion) assays was normalised to the total Cmax of each compound and expressed as a ratio, therapeutic index (TI). The cardiac structural changes of compounds could be divided into four categories: (1) at least one parameter modulated at a concentration of within 10-times the Cmax, (2) at least one parameter modulated at a concentration of within 100-times the Cmax, (3) changes in cardiac structure occurred at concentrations of above 100-times the Cmax concentration, (4) the changes in cardiac structure was not detected in this approach (i.e. no IC_50_ value could be calculated).

A TI of 10-fold was detected for 12 of the 15 structural cardiotoxins and two more if a TI of 100-fold was applied (dasatinib and clozapine; Fig. 6B). The remaining one compound, isoproterenol, was not predicted at therapeutically relevant concentrations of less than 100 times Cmax. Therefore, the greater the safety margin between any *in vitro* cardiac structural changes and the therapeutic exposure, the less likely adverse *in vitro* results will translate to *in vivo* findings.

### Mechanistic fingerprinting of structural cardiotoxicity

The measurement of multiple parameters simultaneously offers the prospect of phenotypic mechanistic insight being gained. To understand the relative contribution of cellular viability (ATP depletion) and imaging parameters (lowest IC_50_), we compared the distribution of each individual compound TI. Our data show that the structural cardiotoxicity of compounds could be associated with four categories: (1) both ATP assay and imaging parameters were predictive, (2) an imaging parameter was most predictive, (3) ATP depletion was most predictive, and (4) cardiotoxicity was not detected by either ATP assay or an imaging parameter at/or below 10-times Cmax concentrations (Fig. 6C). Overall, a combination of imaging parameters and ATP depletion gave greatest sensitivity for the detection of structural cardiotoxicity at therapeutically relevant concentrations. When the TI was considered, no compounds were more sensitive in terms of ATP depletion, whereas sunitinib was more sensitive in an imaging parameter, specifically ER integrity. This illustrates that for some compounds there is differential effects on different parameters, highlighting the possibility that phenotypic mechanistic insight can be gained. To aid this analysis compounds were classified into phenotypic mechanistic fingerprints based on each individual compound response at the lowest therapeutically relevant IC_50_. This approach facilitates the separation of the primary response for a compound from subsequent secondary responses. To compare mechanistic fingerprints across a diverse compound set, the therapeutic total Cmax was again taken into consideration. Responses occurring at concentrations 1, 10 and 100-times the total Cmax concentrations were separated allowing a pathway of changes for each compound to be constructed (fingerprint). Effects occurring at concentrations 10-times or below the total Cmax are potential initiating events in the mechanism of toxicity for these compounds. This analysis revealed multiple fingerprints (Fig. 6D), highlighting structural cardiotoxicity to be multi-factorial and complex in nature. For example, lapatinib, an EGFR and HER2 tyrosine kinase inhibitor, modulated ΔΨm at 1-times or below the total Cmax, this was followed by cellular viability at 10-times or below the total Cmax and lastly ER integrity was modulated at 100-times or below the total Cmax. This data suggests that lapatinib primarily modulates the mitochondria leading to a loss of cell viability with ultimately leads to a decrease in ER integrity. Whereas, the antifungal, amphotericin B, primarily modulated ER integrity, followed by changes in both cellular viability and ΔΨm simultaneously. This suggests ER modulation is the primary organelle modulated in the cardiotoxicity observed. The anthracyclines, doxorubicin and idarubicin, decreased cellular variability followed by a decline in both ΔΨm and ER integrity, suggesting these compounds are grossly cytotoxic.

## Discussion

Cardiovascular diseases are currently recognized as the leading cause of death in the world^1^. Hence it is logical to presume a large proportion of patients receiving novel pharmaceutical agents have preexisting cardiovascular disease^8^. This, combined with improved life expectancy for cancer patients who have been treated with antineoplastic agents associated with the unintended development of chronic heart failure, myocardial infarction, myocarditis and/or cardiomyopathy (drug-induced structural cardiotoxicity), highlights the importance to reduce the cardiovascular burden novel pharmaceutical agents cause. To facilitate a reduction in cardiovascular toxicities associated with novel pharmaceuticals, improved models and approaches are required, particularly for the detection and evaluation of structural cardiotoxicity. Currently structural cardiotoxicity is challenging to monitor clinically prior to the onset of morphological damage and no prognostic biomarkers are available. Current clinical strategies focus on the measurement of LVF and reflective cardiac biomarkers. Several issues surround the use of LVF as a surrogate marker, mainly the dynamic relationship between structural and functional cardiotoxicity. These affects are interlinked, but the sequence and timing is interchangeable and compound specific. Importantly to note, a transient change in LVF without gross pathology is a different magnitude of risk to a compound causing both LVF decline and pathological remodeling/damage. Morphological damage to the heart therefore limits clinical application, progression and use, unless significant benefit is anticipated. The most tangible solution to improve structural cardiotoxicity is the ability to both evaluate and detect this liability early within drug discovery, removing this risk or allowing risk mitigation strategies to be developed. Central to achieving this is improved *in vitro* models. Current models to detect structural cardiotoxicity *in vitro* have various shortcomings that limit their use and impact to support the identification, mitigation and elimination of the risk in drug discovery. Some models rely on the use of ‘cardiac-like’ cell lines with limited morphology and physiology of a cardiomyocyte, for example rat H9c2 cells^19^. Some models use isolated cardiomyocytes from preclinical species, having an impact on animal use. For example, isolated neonatal cardiomyocytes from the mouse and rat^20^. While other models do not take non-myocytes into consideration and solely assess the impact on cardiomyocytes in isolation, for example, monolayer hESC-CMs^18^. Consequently, currently the majority of structural cardiotoxins are first detected in preclinical animal studies.

Our results demonstrate that in a human cardiac microtissue, composed of hiPS-CMs, cardiac endothelial cells and cardiac fibroblasts, we were able to both evaluate and detect structural cardiotoxicity. Via the application of a detailed histopathological and clinical pathology assessment, we have for the first time demonstrated the morphological integrity and cellular composition of these microtissues, confirming their suitability to detect structural cardiotoxins in a higher throughout manner, using imaging and cellular viability endpoints (ΔΨm, ER integrity and ATP depletion). Our immunofluorescence data compares favorably with that obtained from alternative cardiac spheroid models^35–37^. Our data overcomes the limitations associated with other *in vitro* models used to assess structural cardiotoxicity. The high-throughput imaging and cellular viability assessment (validated via detailed morphological assessments) are able to generate hundreds of data points a day, therefore meeting the throughput required to influence chemical design during drug discovery and development; it does not use cells from pre-clinical species or embryonic cells, reducing the use of animals during drug discovery and ethical concerns; and importantly both cardiomyocytes and non-myocytes are present and morphologically intact with key elements of cardiac physiology and morphology being present.

Cardiac microtissues display typical morphological and cellular composition properties to those present in the myocardium and favorable pharmacological responses to known structural cardiotoxins when compared to current approaches. For example, the multi tyrosine kinase inhibitor, sorafenib tosylate, in cardiac microtissues results in a cellular viability (ATP depletion) IC_50_ of 8.72 µM, which compares favorably with the reported IC_50_ value in H9c2 cells and hESC-CMs, 44.89 µM and 15.2 µM, respectively^18,19^. Additionally, our data compares favorably to that obtained in an alternative cardiac spheroids^35,38^. For example, doxorubicin in cardiac microtissues results in a cell viability (ATP depletion) IC_50_ of 15.5 µM following 24 hour exposure, where Polochuk *et al*.^35^ reported a significant change in live/dead staining in the 5–40 µM range and Takeda *et al*.^38^ reported increased LDH release and a decrease in cell viability at 5 and 10 µM. Overall, our data provides evaluations over a full concentration-response range with multiple pharmacological agents.

This is the first study reporting the use of a large, diverse panel of different structural cardiotoxin drug classes in combination with an advanced cardiac *in vitro* model assessing cardiac structure. Studies to date in advanced cardiac models have assessed cardiac function, primarily profiling a small number of compounds^35,39,40^ or specific functional acute alterations^41^. Our data suggests that the combination of imaging parameters and assessment of cellular viability in cardiac microtissues can predict the *in vivo* outcome of structural cardiotoxins (change in cardiac morphology or clinical biomarker release) with an overall sensitivity of 73% and specificity of 86%. Using the published framework to assess the translation of *in vitro* data to findings in *in vivo* models and humans, this approach would be categorized as sufficient to excellent^42,43^. This compares favorably with the predictive value of imaging parameters in hESC-CMs, which have a sensitivity of 74% and specificity of 74%^18^. This highlights that this approach offers improved specificity without a significant compromise on sensitivity. These improvements over stem cell derived cardiomyocyte approaches to evaluate and detect structural cardiotoxicity could be the consequence of several factors. It can be hypothesized that the presence of the additional cell types (cardiac fibroblast and endothelial cells) could be acting on the cardiomyocytes directly or indirectly via altered signaling pathways modulating their function and morphology. Recently, a central role of cardiac fibroblasts in sunitinib cardiotoxicity has been suggested^32^, while endothelial dysfunction has been implicated in doxorubicin structural cardiotoxicity^33^. These emerging findings implicating non-myocytes in structural cardiotoxicity further emphasising the importance of these cell types being present in cardiac *in vitro* models. In addition, these cell types may offer some protection to the cardiomyocytes from gross cellular cytotoxicity potentially contributing to the improved specificity^44^. In addition, unlike monolayer *in vitro* models, the cardiac microtissues are not attached to a solid surface. Consequently, they lack artificial resistance and instead the stiffness in the culture system is generated from cell-cell contact rather than the culture surface. Thus, there are no artificial forces influencing the cardiac microtissues^40,45^. Previous studies investigating structural cardiotoxicity identified the importance of the use of an *in vitro* model that was actively contracting^18^. As these cardiac microtissues have a higher spontaneous beat rate^41^, it could also be hypothesized that the increased contraction, and thus energy utilization, increases the sensitivity to perturbations and that small modifications in the cellular ATP pool could potentially have a greater impact on cellular health in cells with a higher basal beat rate.

The assessment of multiple endpoints simultaneously, and evaluation of the data in the context of the clinical concentration at which structural cardiotoxicity was observed, allows insight into mechanisms of structural cardiotoxins via the development of a unique fingerprint of alterations for each compound. Nevertheless, alterations in one parameter may not be independent or mutually exclusive in the biological sense. For example, changes seen in ΔΨm at lower concentrations may prelude to apoptotic cell death and ATP depletion or loss of ER integrity through changes in ATP utilisation. Conversely, changes in ΔΨm at higher concentrations may drive the accumulation of calcium into the mitochondria from the ER^46–48^. Taken collectively, the changes in imaging parameters and cellular viability facilitates mechanistic insight into the mechanisms of structural cardiotoxicity. It can be hypothesized that changes occurring within 10-times the clinical Cmax represent the primary initiating event in the mechanism of toxicity, whereas those changes occurring at above 10-times the clinical Cmax may represent secondary changes to, in some cases, an unidentified upstream target. Our fingerprint analysis of structural cardiotoxins revealed a diverse array of mechanisms, however either ΔΨm or ER integrity were modulated within 10-fold the clinical total Cmax where cardiac damage has been reported for 12 out of 15 compounds assessed, implicating these organelles to be central to the mechanisms of toxicity. A notable exception was dasatinib, indicating that the mechanism of this structural cardiotoxicity is different from other tyrosine kinase inhibitors inhibiting similar kinases, for example imatinib^49^. This data supports the concept that structural cardiotoxicity is not a class effect and has identified several mechanistic fingerprints. Validating this fingerprint approach, sunitinib was found to modulate ER integrity and subsequently changing both ΔΨm and cellular viability. Sunitinib has previously been implicated in modulating calcium handling in cardiomyocytes, this ultimately impacts the ER^50^. Using this analysis, fingerprints were generated for 14 out of the 15 compounds assessed, however isoproterenol modulated parameters at concentrations above 100-times the clinical total Cmax. One possible explanation for this finding, is in the aetiology of how isoproterenol structural cardiotoxicity develops. Unlike for the other structural cardiotoxins, isoproterenol increases cardiac contractility and heart rate, which if elevated chronically results in pathological lesions in the heart^12^. Therefore, in a model without both pre- and after-load it is not surprising that structural cardiotoxicity resulting from haemodynamic changes is not detected at therapeutically relevant concentrations. This evaluation of mechanistic fingerprints can also be used to provide insights into the potential role of cardiomyocytes and non-cardiomyocytes in structural cardiotoxicity when data is compared to monolayer cardiomyocytes. For example, comparing the mechanistic fingerprints obtained for amphotericin B in hESC-CMs^18^ to those in the cardiac microtissue suggests that either cardiac fibroblasts or endothelial cells are potentially mediating the structural cardiotoxicity. This is apparent by the modulation of ER integrity prior to modulation of both ΔΨm and cellular viability, which could be indicative of cytotoxicity in cardiac microtissues, whereas in hESC-CMs cellular viability was identified as the primary event. This potentially highlights the non-cardiomyocytes as being preferentially modulated. In addition to the identification of mechanisms of toxicity through the presence of non-cardiomyocytes, two compounds (clozapine and cyclophosphamide) are detected in the cardiac microtissue and not hESC-CMs. This again, highlights, the potential role for non-cardiomyocytes in the structural cardiotoxicity of these compounds. Additionally, several synergies between the mechanistic fingerprints obtained in cardiac microtissues and hESC-CMs exist. For example, both amiodarone and bortezomib resulted in the same fingerprint across the two models. This not only suggests that cardiomyocytes are the preferentially modulated cell type, but also provides further evidence for the identified mechanistic fingerprints.

An important consideration with the high-throughput imaging and cellular viability parameters is how these parameters relate to both morphological damage in preclinical animals and clinically in humans. Our data using histopathology and clinical pathology assessments has allowed us to build evidence for the correlation of damage to techniques used in preclinical animal models^51^. However, how these changes related to the clinical manifestation of clinical structural cardiotoxicity characterised by chronic heart failure, myocardial infarction, myocarditis and/or cardiomyopathy remains to be determined.

In conclusion, our data show that cardiac microtissues provide a suitable model for the evaluation and detection of drug-induced structural cardiotoxicity, overcoming the limitations of current approaches and providing a physiological model to determine mechanistic insights. Application in this manner will contribute towards an improved balance between efficacy and safety, reducing the cardiovascular burden of novel pharmaceuticals on patient populations.

## Materials and Methods

### Study design

Cardiac microtissues were developed and morphologically characterized via detailed histopathology and cardiac biomarker assessment. High-throughput imaging parameters and cellular viability assays were developed, allowing the assessment of 15 structural cardiotoxins and 14 non-structural cardiotoxins. All experiments were conducted on a minimum of *n* = 3 microtissues on each independent microtissue preparation. This was conducted on a minimum of three independent microtissue preparations (biological replicates), details in the figure legends.

### Compound preparation

Compound classification of structural cardiotoxins was gathered using Pharmapendium (https://www.pharmapendium.com). Classical terminologies for cardiac injury (i.e. cardiomyopathy, myocarditis, heart failure) were used to identify compounds in Pharmapendium with clinical adverse event reports. In addition, classifications were manually checked for the occurrence of structural cardiotoxicity in the FDA approval package. A compound was classified as a non-structural cardiotoxin if no reports of the above clinical assertions were present in the FDA approval package. Full details of all compounds tested can be found in Supplementary Table 2.

Compounds were initially formulated in dimethyl sulfoxide (DMSO) as a 1000x stock solution (w/v) as previously described^24^. For both microtissues and monocultures, 6 concentrations in a half log_10_ dilution scheme were prepared and diluted 1000x in microtissue media (50% iCell™ cardiomyocytes maintenance media (Cellular Dynamics International (Madison, WI) and 50% endothelial basal medium 2 (PromoCell)). The final DMSO concentration was 0.1% (v/v). Insolubility and precipitation were not seen with any compound (assessed by visual inspection). Compounds were screened in quadruplicate on three separate occasions.

### Cell culture and microtissue formation

hiPS-CMs (iCell™ Cardiomyocytes), cardiomyocyte cell culture thawing and maintenance media were purchased from Cellular Dynamics International (cat# CMM-100-120-005) (Madison, WI). Differentiation protocols and cell culture conditions were as detailed in Ma *et al*. 2011. Primary human cardiac microvascular endothelial cells (hCMECs) and primary human cardiac fibroblasts (hCFs) were obtained from PromoCell (Heidelberg, Germany). hCMECs were cultured in endothelial basal medium 2 (PromoCell, C-22011) supplemented with 2% FCS, EGF (5 ng ml^−1^), bFGF (10 ng ml^−1^), IGF (20 ng ml^−1^), VEGF (0.5 ng ml^−1^), Ascorbic Acid (1 μg ml^−1^) and Hydrocortisone (0.2 µg ml^−1^). hCFs were cultured in fibroblast growth medium 3 (PromoCell, C-23025) supplemented with 10% FCS, bFGF (1 ng ml^−1^) and Insulin (5 µg ml^−1^). Both hCMECs and hCFs (passage <10) were sub-cultured prior to microtissue formation according to supplier’s instructions. Microtissues were formed by combining cell suspensions of hiPS-CM, hCF and hCMEC to give a concentration of 500 cells per 40 μl comprising two-parts hiPS-CM and one-part hCF/hCMEC in thawing microtissue media (50% iCell thawing media, 50% endothelial basal medium 2), resulting in a final cell ratio of 4:2:1 respectively, seeded in 384 well ultra-low attachment spheroid microplates (Corning, 3830)^18^. Cardiac microtissue media was topped up to 80 µl with maintenance microtissue media (50% iCell maintenance media, 50% endothelial basal medium 2) after 48 hours in culture. Microtissues were cultured for a minimum of 14 days prior to experimentation with media exchanges twice per week. A quality control step staining for ΔΨm (TMRE) or F-actin (Phalloidin) was utilised to determine the health status of the cardiac microtissues for each experiment. For all data described in this paper the cardiac microtissues were of good structural integrity at baseline/prior to compound addition.

For 2D monoculture, hiPS-CM, hCMEC and hCF were cultured as described above and seeded in CellCarrier-384 Ultra microplates (Perkin Elmer, 6057300) at 5000, 2500 and 1750 cells/well respectively. Prior to dosing, media was replaced with maintenance microtissue media.

### Histopathology processing, staining and imaging

Microtissues were harvested from 384 well-plates and washed twice in 1x PBS (Sigma P7059) subsequent to fixation in 3.7% paraformaldehyde (PFA) (Sigma F1635) at 4 °C for one hour. The 3.7% PFA was then removed and replaced with two washes of 1x PBS. The PBS was removed and replaced with liquid HistoGel (Thermo Fisher HG-4000-012) and left on ice for 30 minutes to solidify. The HistoGel pellet was placed into a histological cassette and processed using an enclosed tissue processor overnight. The HistoGel was then embedded using paraffin wax.

The formalin fixed paraffin embedded (FFPE) blocks were then sectioned at each time point at three 50 µm step levels with 8 × serial sections per level at 3 µm. H&E staining was performed on microtissue sections using Mayer’s Haematoxylin using a standard automated protocol. Standard automated immunohistochemistry (IHC) and immunofluorescence (IF) protocols were used for primary antibody detection at different dilutions (Supplementary Table 3) on microtissue sections. Diaminobenzadine (DAB) (Roche Diagnostics 760–159) was used to visualise positive staining in single-plex staining and Alkaline Phosphatase Fast Red (Roche Diagnostics 760–160) with DAB were used in dual staining, with a haematoxylin counterstain. IF stained sections were imaged with an Olympus BX61 microscope with the Olympus SC100 camera and cellsSens software. H&E and IHC sections were imaged using the Hamamatsu Nanozoomer 3.1 and Aperio ImageScope software (v12.3.1.6002). A minimum of 15 microtissues per time point/treatment were evaluated. A ‘by-eye’ assessment of all sections was conducted by an experienced toxicological pathologist.

### Immunofluorescence

Cardiac microtissues were harvested, fixed and immunostained as previously described in Ravenscroft *et al*. 2016 with technical modifications. Briefly, microtissues were transferred to 384 well flat-bottom plates and imaged on a Cell Voyager 8000 (Yokogawa Inc.). Images were captured using a 40X water immersion objective (Olympus UPLSAPO 1.0 NA, 0.6 mm WD). Hoescht was imaged using a 405 nm excitation laser (405 ± 5 nm, 100 mW, Coherent) and a Hamamatsu Orca Flash 4 sCMOS camera with 445/45 nm band pass emission filter. Collagen I was imaged using a 488 excitation laser (488 ± 2 nm, 150 mW, Coherent) and a 525/50 nm band pass emission filter. Images were captured over a 100 μm range in the Z-axis with a 2 μm interval between slices. Z-stack images were output as a maximal projection of multiple z-planes.

### Soluble biomarker assessment

Soluble biomarker concentrations were assessed using MILLIPLEX MAP Human Cardiovascular Disease (CVD) Magnetic Bead Panel 1 - Cardiovascular Disease Multiplex Assay. 60 µL of supernatant per microtissue was collected and stored at −80 °C. The assay (3 plex: cTnI, CK-MB and FABP-3) was performed according to manufacturer instructions. Fluorescence intensity was obtained using Bio-Plex^®^3D instrument and Luminex xPONENT software version 4.2. Briefly, the multiplexed assay uses internally colour–coded 6.45 µm magnetic microspheres which are coated with specific capture antibodies to cTnI, CK-MB or FABP-3. The analyte from the test sample is captured by the coated microspheres. The immuno-complex is incubated with biotinylated antibody and detected using Streptavidin-PE conjugate (reported molecule). Each individual colour coded microsphere is identified using Bio-Plex^®^3D technology and the result of its bioassay is quantified based on fluorescent reporter signals. Bio-Plex Manager software version 6.1 was used to generate standard curves (4 parameter logistic model/5 parameter logistic model) and the biomarker concentration was derived from appropriate standards. The standard curve for each biomarker was generated using lyophilised standards. Samples (including standards and QCs) were run in duplicate and mean fluorescence intensity (MFI) was used to calculate the observed concentration.

### Gene expression analysis

Microtissues were pooled, allowed to pellet by gravity and washed in 1x PBS before lysis with RLT buffer (Qiagen). Total RNA was extracted using an RNeasy Plus Mini Kit (Qiagen). A NanoDrop spectrophotometer (NanoDrop Technologies) was used to quantify RNA samples. 80 ng RNA was reverse transcribed using SuperScript® VILO IV Master Mix (Thermo Fisher Scientific). qRT-PCR was performed using 2 μl of cDNA, TaqMan® Gene Expression Master Mix and the TaqMan® Gene Expression probes of interest (Supplementary Table 4). Amplification was monitored under standard cycling conditions using a 7900 HT Fast Real-Time PCR System (Applied Biosystems, Foster City, CA, U.S.A.). Data was normalised to the geometric mean of two stable housekeeping genes, GAPDH and RPL37A. Relative quantification of gene expression was performed using the ΔΔCt relative quantitation method. The mean Ct value was calculated for each gene and normalized to the endogenous control.

### Confocal imaging: high content biology

Cardiac microtissues were stained with fluorescent probes in cell culture media, 40 µl media was removed and 40 µl of 2x multiplexed dye was added back to the well for 30 minutes at 37 °C, 5% CO_2_. Fluorescent probes used were ER-Tracker blue (2 µM) to measure endoplasmic reticulum (ER) integrity and TMRE (500 nM) to measure mitochondrial membrane potential (ΔΨm). All microtissues were imaged on a Cell Voyager 7000 (YokogawaInc.) and images captured using a 20x objective (Olympus UPLSAPO 0.75 NA, 0.6 mm WD), with a 2 × 2 bin. Plates are imaged live, prior to use the stage temperature was set to 37 °C and CO_2_ level at 5%. ER-Tracker was imaged using a 405 nm excitation laser (405 ± 5 nm, 100 mW, Coherent) and an Andor Neo sCMOS camera with 445/45 nm band pass emission filter. TMRE was imaged using a 561 nm excitation laser (561 ± 2 nm, 200 mW, Coherent) and a 600/37 nm band pass emission filter. Transmitted light images were acquired using a 100 W Halogen lamp as an illumination source. Images were captured over a 60 μm range in the Z-axis with a 5 μm interval between slices. Z-stack images were output as a maximal projection of multiple z-planes.

Monoculture cells were stained with fluorescent probes diluted in cell culture media, 25 µl media was removed and 25 µl of 2x multiplexed dye was added back to the well for 15 minutes at 37 °C, 5% CO_2_. Images were captured at a single plane using the protocol described above.

### Image analysis of cardiac microtissues

Maximum projection images were imported and analysed in the Columbus Platform (v2.7, Perkin Elmer Inc.). Objects were identified using the transmitted light image and the object of interest selected based on morphological and intensity based features from the fluorescence channels. Quantitative measurements of morphological, texture and intensity features from the microtissues were captured for all parameters and exported for data analysis.

### Image analysis of cardiac monocultures

Similarly, for monoculture cells images were imported and analysed as above. Cell masks were created to identify the objects of interest (individual cells) which were selected based on morphological and intensity based features from the fluorescence channels. Quantitative measurements of average fluorescence intensity were captured for all parameters and exported for data analysis.

### Measurement of cellular ATP content

Cellular ATP concentrations were assessed using the CellTiter-Glo^®^ Luminescent Cell Viability Assay as per the manufacturer’s instructions (Promega); for cardiac microtissues 40 µl media was removed prior to the addition of 40 µl CellTiter-Glo^®^ reagent. For monocultures, 25 µl of media was removed and 25 µl of CellTiter-Glo^®^ reagent added to the well. This assay is a luminescence method based on the luciferase/luciferin reaction which determines the number of viable cells present in a well, based on intracellular ATP levels^52^. Data from the same treatment on minimum of 3 occasions were averaged to represent the mean ATP measurement.

### Data analysis

Data produced were analysed using Genedata Screener 13 (Genedata AG, Basel, Switzerland). In brief, all values scaled relative to the median of the negative (0.1% DMSO, (v/v)) control wells. The median of the negative control wells was set at 0 and the signals from all wells scaled to this value. Calculations were performed with all plates separately to balance out trends and to ensure cross plate comparability. Concentration-effect curves were plotted in Screener 13 using an algorithm that excludes outliers and determines an optimal fit using a four-parameter logistic function. The molar concentration of test compound producing 50% inhibition (IC_50_) were calculated for each test compound in a minimum of triplicate and geometric means and 95% CI were subsequently calculated. Receiver operator characteristic (ROC) curves were plotted using GraphPad Prism™ (La Jolla, CA). Therapeutic index (TI) was calculating by dividing the obtained IC_50_ by each compound total Cmax concentration. T-tests to compare the conditions were performed using Graphpad Prism™ 7 (La Jolla, CA).

For soluble biomarker data the median concentration or mean fold change relative to vehicle was calculated from at least three microtissues tested at each condition. Values below the lower limit of quantitation (BLOQ) were inputted as half of lower limit of quantitation (LLOQ/2)^53^. The mean assay LLOQ was used where samples analysis was performed in multiple technical occasions. Fold change relative to vehicle (F) was calculated as the ratio between the biomarker concentration of compound treated (R1) and non-treated (R2) microtissues where F = (R1/R2) − 1. Where F resulted as <0, it was reported as 0. Two-tailed, Mann-Whitney (non-parametric) or unpaired t-test (parametric) was used to test significance. Welch’s correction was used where the variance was not equivalent between groups.

## Electronic supplementary material

Supplementary information
