# Supplementary material for: Characterization and Validation of a Human 3D Cardiac Microtissue for the Assessment of Changes in Cardiac Pathology
Source: Sci Rep. 2018 Jul 5;8:10160. doi: 10.1038/s41598-018-28393-y (PMC6033897; doi:10.1038/s41598-018-28393-y)
Supplement: Supplementary file 1 — Supplementary information [file 41598_2018_28393_MOESM1_ESM.pdf]

CHARACTERIZATION AND VALIDATION OF A HUMAN 3D CARDIAC MICROTISSUE FOR THE ASSESSMENT OF CHANGES IN CARDIAC PATHOLOGY

Caroline R. Archer[1], Rebecca Sargeant[2], Jayati Basak[1], James Pilling[3], Jennifer R. Barnes[2], Amy Pointon[1*]

Safety and ADME Translational Sciences Department, Drug Safety and Metabolism[1], Pathalogy Sciences, Drug Safety and Metabolism[2], Discovery Sciences[3], IMED Biotech Unit, AstraZeneca, Cambridge, UK, CB4 0WG

			24 hours						72 hours					
			ER integrity		Mitochondrial membrane potential (ΔΨm)		Cellular viability (ATP depletion)		ER integrity		Mitochondrial membrane potential (ΔΨm)		Cellular viability (ATP depletion)	
**Compound name**	**Top Concentration Tested (µM)**	**Cell Type**	**IC_{50} (µM)**	**95% confidence interval**	**IC_{50} (µM)**	**95% confidence interval**	**IC_{50} (µM)**	**95% confidence interval**	**IC_{50} (µM)**	**95% confidence interval**	**IC_{50} (µM)**	**95% confidence interval**	**IC_{50} (µM)**	**95% confidence interval**
Doxorubicin HCl	50	hiPS-CM	10.50	4.17	8.28	8.45	9.26	9.86	76.52	20.7	16.87	3.4	1.01	0.8
		hCMEC	43.65	7.31	50	-	1.03	31.99	31.00	11.12	50	-	0.10	0.14
		hCF	6.39	29.51	50	-	0.62	0.43	46.58	6.25	50	-	0.11	0.18
Lapatinib	100	hiPS-CM	23.55	57.78	31.95	53.16	100	-	3.10	5.87	23.56	58.01	100	-
		hCMEC	26.68	10.62	5.47	10.65	16.19	14.78	4.40	3.38	11.59	4.22	3.07	2.77
		hCF	14.71	3.82	42.89	47.05	21.58	4.25	10.99	22.36	28.79	55.03	11.04	3.51
Sunitinib Malate	30	hiPS-CM	4.48	18.47	16.08	14.60	24.70	5.75	2.63	12.63	16.24	12.92	27.09	5.16
		hCMEC	2.96	10.61	9.57	10.18	4.90	7.62	12.73	14.79	2.97	1.86	0.80	0.84
		hCF	6.59	17.37	17.78	13.20	16.46	16.36	0.37	0.15	11.60	12.72	4.00	2.50

Supplementary Table 1: Summary of IC_{50} data of cellular viability (ATP depletion), ER integrity and ΔΨm for 2D monocultures of hiPS-CM, hCMEC or hCF treated with tool structural cardiotoxins for 24 or 72 hours.

								ER integrity		Mitochondrial membrane potential (ΔΨm)		Cellular viability (ATP depletion)	
**Compound number**	**Compound name**	**Cmax (μM)**	**Classification**	**Primary pharmacology**	**FDA approval package cardiac warnings**	**Structural cardiotoxicty literature reference**	**Top concentration tested (μM)**	**IC_{50} (μM)**	**95% confidence interval**	**IC_{50} (μM)**	**95% confidence interval**	**IC_{50} (μM)**	**95% confidence interval**
1	Amiodarone HCl	3.02	Structural cardiotoxin	Antiarrhythmic	Arrhythmia, heart block, sinus bradycardia, CHF, ventricular fibrillation	*1*	50	23.62	1.52	5.29	23.70	15.99	36.03
2	Amphotericin B	9.00	Structural cardiotoxin	Antifungal	Arrhythmia, atrial fibrillation, bradycardia, cardiac arrest, cardiomegaly	*2*	50	3.56	25.52	>50		37.58	12.14
3	Bortezomib	0.29	Structural cardiotoxin	Antineoplastic	CHF, decreased LVEF, isolated cases of QT-interval prolongation	*3*	100	91.77	12.11	1.62	0.41	0.35	22.08
4	Clozapine	0.47	Structural cardiotoxin	Antipsychotic	MI, myocarditis, arrhythmia	*4*	100	7.82	50.40	56.35	38.61	>100	
5	Cyclophosphamide	153.20	Structural cardiotoxin	Antineoplastic	Acute cardiac toxicity, CHF, myocarditis, myocardial necrosis	*5*	100	60.71	35.72	65.67	32.18	54.44	4.45
6	Dasatinib	0.72	Structural cardiotoxin	Antineoplastic	QT prolongation, CHF, LVD and MI, cardiomyopathy, arrhythmia, cardiomegaly	*6*	100	>100		>100		27.14	46.00
7	Doxorubicin HCl	15.34	Structural cardiotoxin	Antineoplastic	CHF, decreased LVEF, sinus tachycardia, myocarditis, cardiomyopathy	*7*	50	3.18	1.48	0.18	0.08	0.83	0.38
8	Fluorouracil	4.61	Structural cardiotoxin	Antineoplastic	HF, MI, ventricular dysfunction, cardiac fibrillation, arrhythmia	*8, 9*	100	60.03	36.19	11.27	46.74	27.53	6.47
9	Idarubicin HCl	0.12	Structural cardiotoxin	Antineoplastic	CHF, arrhythmia, cardiomyopathy, decreased LVEF	*10*	100	1.67	0.52	0.97	1.95	0.03	4.48
10	Imatinib Mesylate	3.54	Structural cardiotoxin	Antineoplastic	CHF, decreased LVEF	*11*	50	>50		6.29	0.71	15.82	47.08
11	Isoproterenol HCl	0.01	Structural cardiotoxin	Bronchodilator	Tachycardia, palpitations, ventricular	*12*	100	76.85	29.13	59.25	36.72	>100	

								ER integrity		Mitochondrial membrane potential (ΔΨm)		Cellular viability (ATP depletion)	
**Compound number**	**Compound name**	**Cmax (μM)**	**Classification**	**Primary pharmacology**	**FDA approval package cardiac warnings**	**Structural cardiotoxicty literature reference**	**Top concentration tested (μM)**	**IC_{50} (μM)**	**95% confidence interval**	**IC_{50} (μM)**	**95% confidence interval**	**IC_{50} (μM)**	**95% confidence interval**
					arrhythmias, myocarditis								
12	Lapatinib	4.18	Structural cardiotoxin	Antineoplastic	Decreased LVEF and HF, QT interval prolongation	*6*	100	>100		2.84	5.13	12.90	41.38
13	Mitoxantrone diHCl	3.31	Structural cardiotoxin	Antineoplastic	CHF, decreased LVEF, tachycardia, arrhythmia	*13*	100	1.10	0.15	0.98	0.07	0.29	21.13
14	Sorafenib Tosylate	16.57	Structural cardiotoxin	Antineoplastic	Cardiac ischemia, MI, QT-interval prolongation (rare)	*6*	100	6.42	56.35	8.22	0.84	8.72	44.79
15	Sunitinib Malate	0.25	Structural cardiotoxin	Antineoplastic	Decreased LVEF and HF, QT interval prolongation and TdP, cardiomyopathy	*14*	30	0.39	0.04	2.58	0.46	8.23	4.93
16	Acyclovir	6.66	Non-structural cardiotoxins	Antiviral	No report		100	>100		>100		>100	
17	Buspirone HCl	0.03	Non-structural cardiotoxins	Antipsychotic	Nonspecific chest pain		100	>100		>100		56.36	2.97
18	Cisapride Hydrate	0.17	Non-structural cardiotoxins	Gastroprokinrtic	Arrhythmia and long QT syndrome		100	32.83	50.48	>100		76.35	7.64
19	Donepezil HCl	0.20	Non-structural cardiotoxins	Dementia	Bradycardia		30	>30		>30		12.38	8.50
20	Erlotinib	3.00	Non-structural cardiotoxins	Antineoplastic	No report		30	>30		>30		20.82	11.41
21	Gemfibrozil	99.87	Non-structural cardiotoxins	Fibrate	No report		100	>100		>100		>100	
22	Ketoprofen	78.65	Non-structural cardiotoxins	Nonsteroidal anti-inflammatory	No report		100	>100		>100		37.64	8.32
23	Mebendazole	2.03	Non-structural cardiotoxins	Antihelmintic	No report		50	5.97	1.07	>50		>50	
24	Methapyrilene	15.30	Non-structural cardiotoxins	Antihistamine	No report		100	75.04	24.72	>100		>100	

								ER integrity		Mitochondrial membrane potential (ΔΨm)		Cellular viability (ATP depletion)	
**Compound number**	**Compound name**	**Cmax (μM)**	**Classification**	**Primary pharmacology**	**FDA approval package cardiac warnings**	**Structural cardiotoxicty literature reference**	**Top concentration tested (μM)**	**IC_{50} (μM)**	**95% confidence interval**	**IC_{50} (μM)**	**95% confidence interval**	**IC_{50} (μM)**	**95% confidence interval**
25	Minoxidil	14.81	Non-structural cardiotoxins	Antihypertensive	Rapid heart beat		50	>50		>50		>50	
26	Narigenin	15.40	Non-structural cardiotoxins	Anti-inflammatory	No report		100	>100		>100		62.46	7.94
27	Nifedipine	0.58	Non-structural cardiotoxins	Anti-hypertensive	Hypertension, angina		100	35.61	1.05	>100		43.95	19.00
28	Praziquantel	0.64	Non-structural cardiotoxins	Antihelmintic	No report		100	0.82	0.16	>100		10.14	2.81
29	Terfenadine	0.13	Non-structural cardiotoxins	Antihistamine	TdP		100	23.65	1.15	>100		13.04	10.31

Supplementary Table 2: Summary of compound classification, total Cmax and IC_{50} data for cellular viability (ATP depletion), ER integrity and ΔΨm.

Antibodies	Dilution	Manufacturer	Catalogue No.
Rabbit Polyclonal anti-Caspase 3 (IHC)	1:1000	Abcam	ab13847
Rabbit Polyclonal anti-CD31 (IHC)	1:50	Abcam	ab28364
Rabbit Monoclonal anti-Sarcomeric Alpha Actinin (IHC)	1:500	Abcam	ab68167
Rabbit Polyclonal anti-Cardiac Troponin I (IHC)	1:200	Abcam	ab47003
Mouse monoclonal anti Ki67 (IHC)	1:50	Dako	F726801-8
Rabbit Polyclonal anti-Cardiac Troponin I (IF)	1:10	Abcam	ab47003
Rabbit Monoclonal anti-Vimentin (IHC)	1:2000	Abcam	ab92547
Mouse Monoclonal anti-Vimentin (IF)	1:200	Dako	M0725
Rabbit Polyclonal anti-Collagen I (IF)	1:500	Abcam	ab34710
Chicken anti-Rabbit Alexa Fluor 594 (IF)	1:200	Thermo Fisher	A21442

Supplementary Table 3: Antibody information for IF and IHC.

List of primary and secondary antibodies used to analyse cardiac microtissues in both IF and IHC experiments.

Marker type	Gene	TaqMan® Assay ID
Housekeeping	*GAPDH*	Hs99999905_m1
	RPL37A	Hs01102345_m1
Biomarker	*FABP3*	Hs00997360_m1
	CKM	Hs00176490_m1
	CKB	Hs00176484_m1
	TNN3I	Hs00165957_m1
Structural	*COL1A1*	Hs00164004_m1

Supplementary Table 4: qRT-PCR TaqMan® Gene Expression assay information

List of qRT-PCR TaqMan® assays used to analyse gene expression in cardiac microtissues.

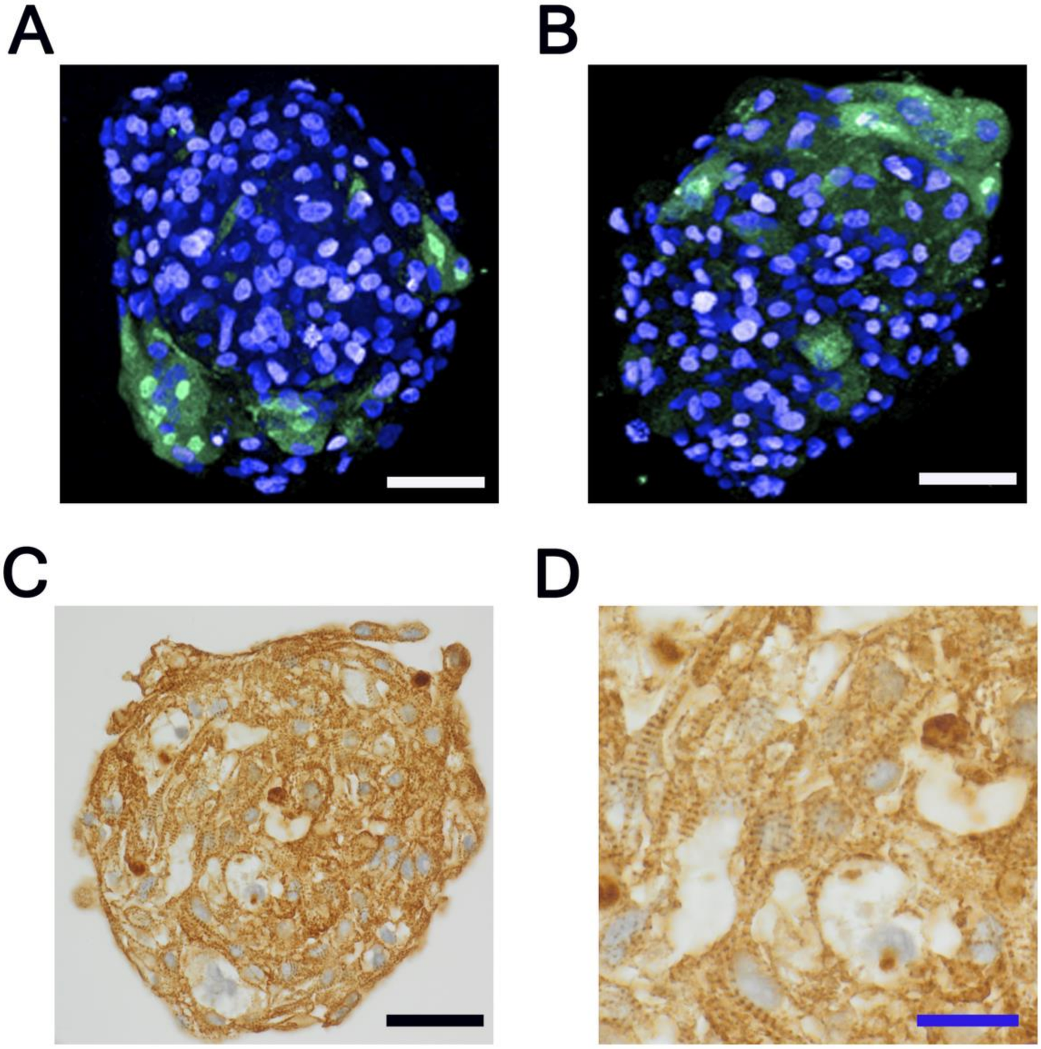

Supplementary Figure 1: Cellular composition of cardiac microtissues. Representative immunofluorescence (IF) images of cardiac microtissues immunostained with Collagen I (green) and Hoechst 33342 (blue) staining the nucleus at Day 14 (**A**) and Day 21 (**B**). (**C** and **D**) Representative immunohistochemistry (IHC) images of microtissues immunostained with alpha-actinin (α-actinin). White and black scale bar represents 50 μm, blue scale bar represents 12.5 μm.

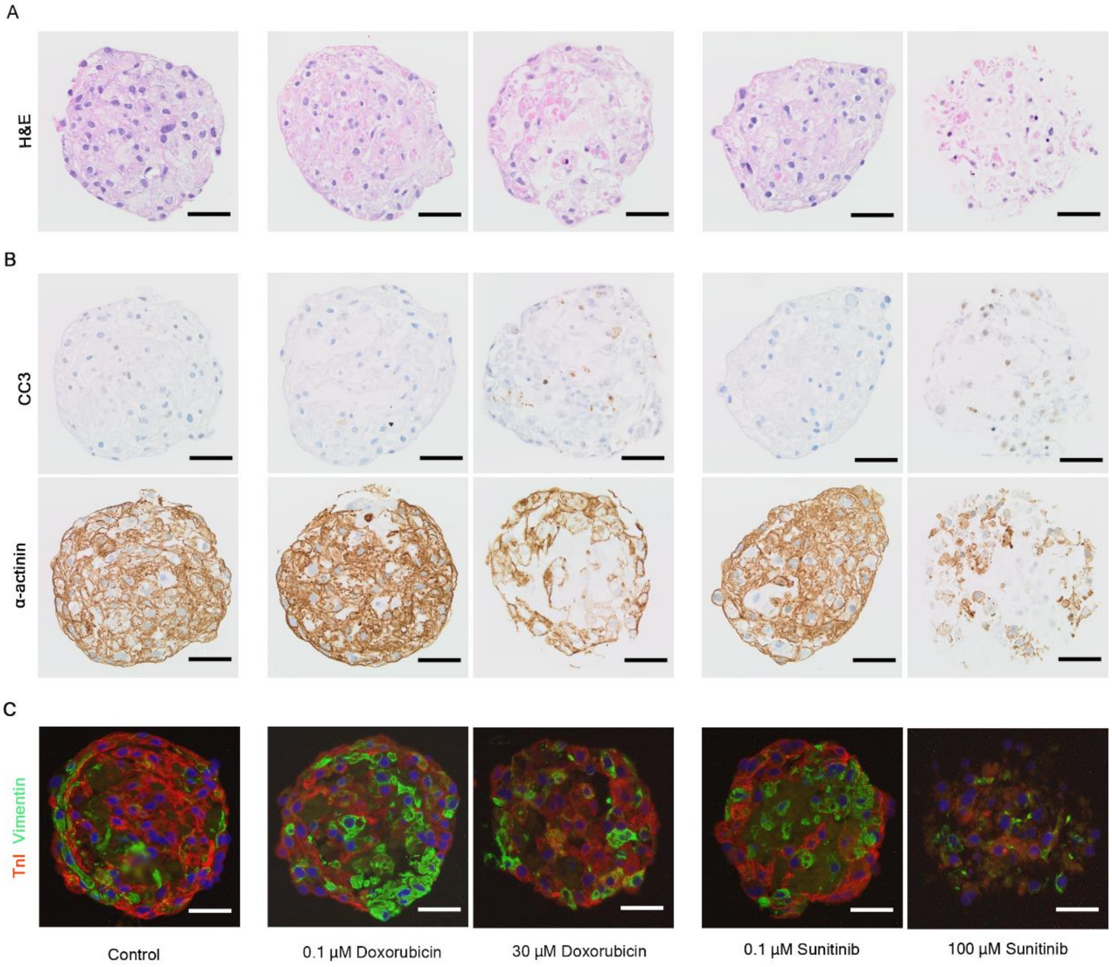

Supplementary Figure 2: Treatment with known structural cardiotoxins for 24 hours results in significant histopathological changes and loss of key structural proteins in a concentration dependent manner. Microtissues were exposed to 0.1 and 100 µM sunitinib and 0.3 and 30 µM doxorubicin (**A**) Representative images of H&E stained microtissues. (**B**) Representative IHC images showing an increase in CC3 expression in drug treated microtissues. Representative IHC and IF images showing the reduced expression of the cardiomyocyte structural proteins α-actinin (**B**) and cTnI (**C**), respectively. Scale bar represents 50 µm. All morphology data evaluated a minimum of 15 microtissues per time point.

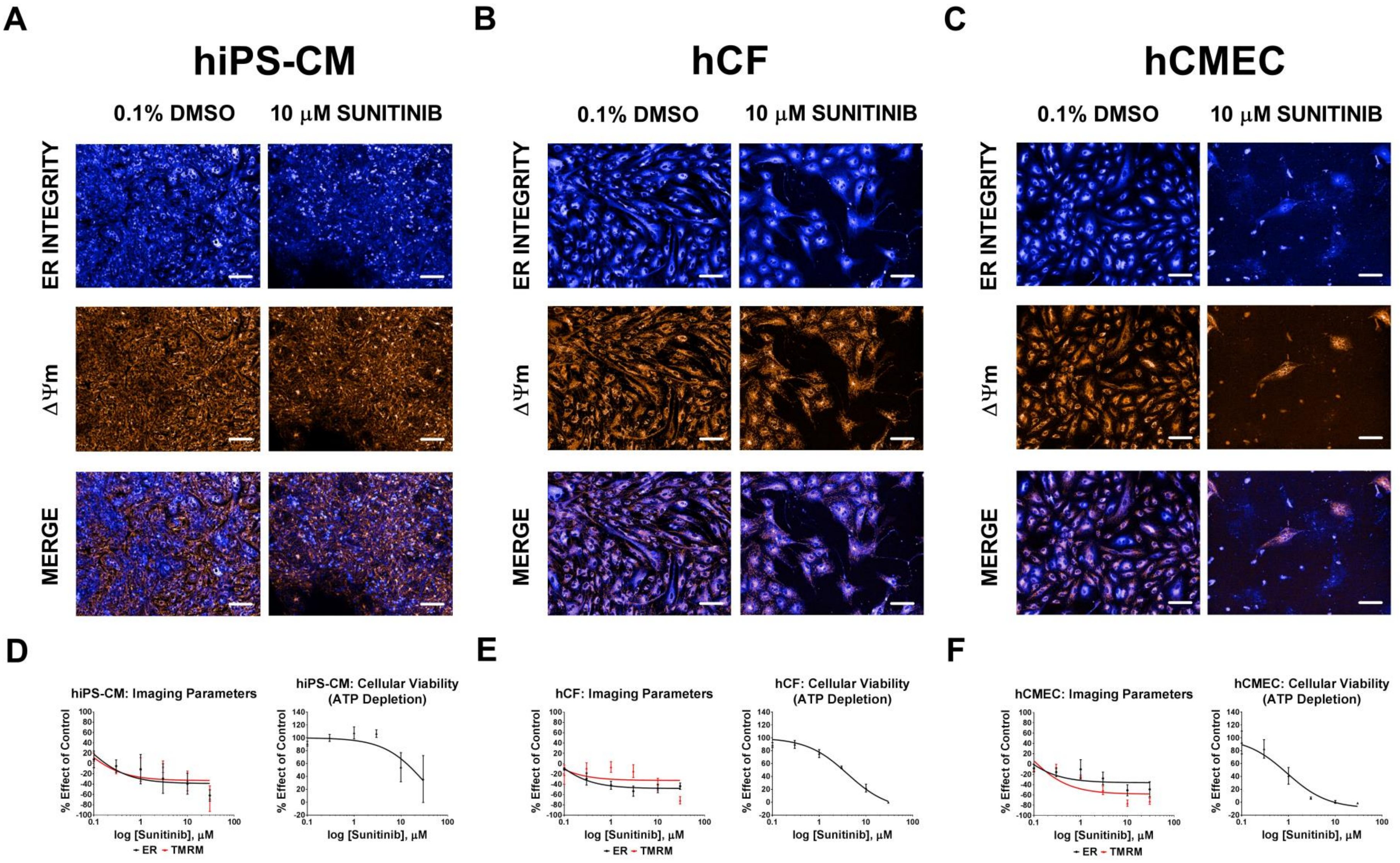

Supplementary Figure 3: Validation of HCB structural cardiotoxicity end points following treatment with sunitinib for 72 hours. Representative fluorescent image of monocultures of hiPS-CM (**A**), hCF (**B**) or hCMEC (**C**) treated with 0.1 % DMSO or 10 µM sunitinib for 72 hours. Monocultures (**A-C**) stained with ER tracker (ER integrity) or TMRM (mitochondrial membrane potential; ΔΨm). Scale bar represents 100 µm.

Dose response curves of Imaging parameter (ER Integrity and ΔΨm) or cellular viability (ATP depletion) for hiPS-CM (**D**), hCF (**E**) and hCMEC (**F**) treated with 10 µM sunitinib for 48 hours (*n* = 3, mean ± SEM). ΔΨm; mitochondrial membrane potential.

References

1. Dykens, J. A. & Will, Y. The significance of mitochondrial toxicity testing in drug development. *Drug Discov Today* **12**, 777-785, (2007).

2. Danaher, P. J., Cao, M. K., Anstead, G. M., Dolan, M. J. & DeWitt, C. C. Reversible dilated cardiomyopathy related to amphotericin B therapy. *J Antimicrob Chemother* **53**, 115-117, (2004).

3. Nowis, D. *et al.* Cardiotoxicity of the anticancer therapeutic agent bortezomib. *Am J Pathol* **176**, 2658-2668, (2010).

4. Layland, J. J., Liew, D. & Prior, D. L. Clozapine-induced cardiotoxicity: a clinical update. *Med J Aust* **190**, 190-192 (2009).

5. Floyd, J. D. *et al.* Cardiotoxicity of cancer therapy. *J Clin Oncol* **23**, 7685-7696, (2005).

6. Force, T., Krause, D. S. & Van Etten, R. A. Molecular mechanisms of cardiotoxicity of tyrosine kinase inhibition. *Nat Rev Cancer* **7**, 332-344, (2007).

7. Minotti, G., Menna, P., Salvatorelli, E., Cairo, G. & Gianni, L. Anthracyclines: molecular advances and pharmacologic developments in antitumor activity and cardiotoxicity. *Pharmacol Rev* **56**, 185-229, (2004).

8. Schimmel, K. J., Richel, D. J., van den Brink, R. B. & Guchelaar, H. J. Cardiotoxicity of cytotoxic drugs. *Cancer Treat Rev* **30**, 181-191, (2004).

9. Bocci, G. *et al.* Comparative pharmacokinetic analysis of 5-fluorouracil and its major metabolite 5-fluoro-5,6-dihydrouracil after conventional and reduced test dose in cancer patients. *Clin Cancer Res* **6**, 3032-3037 (2000).

10. Anderlini, P. *et al.* Idarubicin cardiotoxicity: a retrospective study in acute myeloid leukemia and myelodysplasia. *J Clin Oncol* **13**, 2827-2834, (1995).

11. Kerkela, R. *et al.* Cardiotoxicity of the cancer therapeutic agent imatinib mesylate. *Nat Med* **12**, 908-916, (2006).

12. Zhang, X., Wei, M., Zhu, W. & Han, B. Combined transplantation of endothelial progenitor cells and mesenchymal stem cells into a rat model of isoproterenol-induced myocardial injury. *Arch Cardiovasc Dis* **101**, 333-342, (2008).

13. Kingwell, E. *et al.* Cardiotoxicity and other adverse events associated with mitoxantrone treatment for MS. *Neurology* **74**, 1822-1826, (2010).

14. Chu, T. F. *et al.* Cardiotoxicity associated with tyrosine kinase inhibitor sunitinib. *Lancet* **370**, 2011-2019, (2007).
